# Enhancing biofeedback-driven self-guided virtual reality exposure therapy through arousal detection from multimodal data using machine learning

**DOI:** 10.1186/s40708-023-00193-9

**Published:** 2023-06-21

**Authors:** Muhammad Arifur Rahman, David J. Brown, Mufti Mahmud, Matthew Harris, Nicholas Shopland, Nadja Heym, Alexander Sumich, Zakia Batool Turabee, Bradley Standen, David Downes, Yangang Xing, Carolyn Thomas, Sean Haddick, Preethi Premkumar, Simona Nastase, Andrew Burton, James Lewis

**Affiliations:** 1 Department of Computer Science, Nottingham Trent University, Clifton Lane, Nottingham, NG11 8NS UK; 2 Medical Technologies Innovation Facility, Nottingham Trent University, Clifton Lane, Nottingham, NG11 8NS UK; 3 Computing and Informatics Research Centre, Nottingham Trent University, Clifton Lane, Nottingham, NG11 8NS UK; 4 School of Social Sciences, Nottingham Trent University, Shakespeare St, Nottingham, NG1 4FQ UK; 5 Nottingham School of Art & Design, Nottingham Trent University, Shakespeare St, Nottingham, NG1 4FQ UK; 6 School of ADBE, Nottingham Trent University, Shakespeare St, Nottingham, NG1 4FQ UK; 7 Division of Psychology, London South Bank University, London, SE1 0AA UK; 8 Independent Clinical Psychologist, London, UK

**Keywords:** Biofeedback, Arousal, EEG, HRV, Glossophobia, Stress, VRET

## Abstract

Virtual reality exposure therapy (VRET) is a novel intervention technique that allows individuals to experience anxiety-evoking stimuli in a safe environment, recognise specific triggers and gradually increase their exposure to perceived threats. Public-speaking anxiety (PSA) is a prevalent form of social anxiety, characterised by stressful arousal and anxiety generated when presenting to an audience. In self-guided VRET, participants can gradually increase their tolerance to exposure and reduce anxiety-induced arousal and PSA over time. However, creating such a VR environment and determining physiological indices of anxiety-induced arousal or distress is an open challenge. Environment modelling, character creation and animation, psychological state determination and the use of machine learning (ML) models for anxiety or stress detection are equally important, and multi-disciplinary expertise is required. In this work, we have explored a series of ML models with publicly available data sets (using electroencephalogram and heart rate variability) to predict arousal states. If we can detect anxiety-induced arousal, we can trigger calming activities to allow individuals to cope with and overcome distress. Here, we discuss the means of effective selection of ML models and parameters in arousal detection. We propose a pipeline to overcome the model selection problem with different parameter settings in the context of virtual reality exposure therapy. This pipeline can be extended to other domains of interest where arousal detection is crucial. Finally, we have implemented a biofeedback framework for VRET where we successfully provided feedback as a form of heart rate and brain laterality index from our acquired multimodal data for psychological intervention to overcome anxiety.

## Introduction

Anxiety is an emotional state characterised by negative affect and worry, heightened arousal, careful environmental monitoring, rumination and avoidance behaviour, ranging from mild to severe. Intense states of anxiety, or even fear—a more rudimentary physiological response to a perceived threat that can lead to fight/flight/freeze reactions and panic behaviour—can be symptoms of different psychological disorders. For example, phobias are defined by an exaggerated fear or unrealistic sense of threat to a situation or object, which appear in many forms. In the Diagnostic and Statistical Manual of Mental Disorders (DSM-5, 2013) [1, 2], the American Psychiatric Association defines five types of phobia, related to natural environments (e.g., heights), animals (e.g., spiders), specific situations (e.g., public spaces), blood/injury or medical issues, and other types (e.g., loud noise, vomiting, choking). These debilitating disorders affect about 13% of the world’s total population. Research is ongoing for contributing factors to the onset, development, and maintenance of phobias and anxiety-related disorders, their underlying cognitive and behavioural processes, physical manifestation, and treatment methods [3]. Traditional treatments of such disorders include in vivo exposure, interoceptive exposure, cognitive behavioural therapy (CBT), applied muscle tension, supportive psychotherapy, hypnotherapy, and medications such as beta-blockers or sedatives [4].

Virtual reality exposure therapy (VRET) is one of the most promising novel treatments, enabled by its superior immersive capabilities that generate a greater sense of presence and enhance user effects, especially for negatively valenced, high arousal stimuli [5]. Over the last two decades VRET, encompassing psychological treatment principles and enabled by advancing display and computing technology developments, has become a popular digital intervention for various psychological disorders [6, 7], being as effective as in vivo (i.e., face-to-face) exposure therapy post-intervention [8]. For example, a meta-analysis showed VRET for Social Anxiety Disorder (encompassing an exaggerated fear of being rejected, negatively evaluated or humiliated during social interactions, observations and/or in performance situations) to be more effective than wait-list controls (with large effect sizes), and even therapist-led in vivo exposure therapy (though only small effect size) [6]. It shows good acceptability in users due to its safe, controlled and empowering means of exposure. The state-of-the-art development clearly lacks one key development; there is no attempt of real-time biofeedback for VRET intervention. A vital part of our development of VRET is the integration of bio-signals, such as heart rate variability or cortical arousal, to assess and ameliorate physiological distress states (e.g., fear or anxiety-induced arousal) during exposure. Here, the correct detection of physiological states through robust models for the effective management of anxiety-induced arousal or stress is pivotal to facilitating intervention and enhancing psychological health and well-being. However, a reliable and automated system is needed to accomplish this task. Given that artificial intelligence (AI) and machine learning (ML) have been playing significant roles in the methodological developments for diverse problem domains, including computational biology [9, 10], cyber security [11–14], disease detection [15–21] and management [22–27], elderly care [28, 29], epidemiological study [30], fighting pandemic [31–37], healthcare [38–42], healthcare service delivery [43–45], natural language processing [46–50], social inclusion [51–53] and many more, the AI and ML-based methods can be employed to do this task. Hence, here we have explored a series of ML models with publicly available data sets (using electroencephalogram and heart rate variability) to predict arousal states. If we can detect anxiety-induced arousal, we can trigger calming activities to allow individuals to cope with and overcome distress. Here, we discuss the means of effective selection of ML models and parameters in arousal detection. We have presented our first abstract concept ML Driven Self-guided Virtual Reality Exposure Therapy Based on Arousal State Detection from Multimodal Data in [54]. Then we started implementation, and here in this paper, we have added the concept of Biofeedback as a form of variation of heart rate and laterality index using EEG data and synthesised heart rate collected by emotive EPOC flex [55].

## Related work

Arousal detection, a noninvasive intervention, requires a multi-disciplinary approach, where psychological state determination, ML models for arousal or stress detection, and exploration of the related domains for model implementation are equally important. In this paper, we narrow down the areas and present an overview of the state-of-the-art scenarios.

### Emotion/stress detection

Koelstra et al. [56] presented a multimodal dataset for the analysis of human affective states. They collected physiological signals, including electroencephalographic (EEG) data from participants watching music videos and rated each video in terms of excitement, stress, arousal, flaws, valence, like, dislike. The data has been widely used for developing various ML models for arousal, anxiety and stress detection. Ahuja and Banga [57] created another dataset where they classified mental stress in 206 students. They used linear regression (LR), support vector machine (SVM), Naïve Bayes (NB) and random forest (RF) ML classification algorithms [9, 30, 38, 41, 49, 51, 58–60] to determine mental stress. Using SVM and tenfold cross-validation, they claimed an 85.71% accuracy. Ghaderi et al. [61] used respiration, galvanic skin response (GSR) from hand and foot, heart rate (HR) and electromyography (EMG) at different time intervals to examine different stress levels. Then they used k-nearest neighbour (*k*-NN) and the SVM ML model for stress detection [61].

**Table 1:** Machine learning models of arousal detection

Refs.	Domain	Data type	Model	Performance	Modality
[3]	Acrophobia	GSR, HR, BVR	SVM, RF, *k*-NN	SVM-42.6%, *k*-NN-89.5%, RF-99%	Unimodal
[62]	Drug addiction	HRV	PCA, *k*-Means++	.	Unimodal
[63]	Spider phobia	Clinical characteristics	RF, Permutation Test	$$*p < 0.05$$; $$**p < 0.01$$; $$***p < 0.001$$	Unimodal
[64]	Spider phobia	fMRI, genetic data	SVM, GPC	.	Unimodal
[65]	PSA	.	.	.	Unimodal
[66]	Anxiety disorder	EEG	SVM	Healthy subjects-97.70 ± 3.32%, Anxious subjects-92.29 ± 4.44%	Unimodal
[67]	Stress	EEG	*k*-NN with GA-based feature selection	k-NN 71.76%	Unimodal
[68]	Emotion recognition	EEG	SVM, RF	RF-74.0%, SVM-57.2%	Unimodal
[69]	Major depressive disorder	EEG	*k*-NN, SVM, CNN	CNN-94.13%, SVM-88.22%, *k*-NN-83.15%	Unimodal
[70]	Stress	EEG	SVM, NB	SVM-90%, NB-81.7%	Unimodal
[56]	Human affective state	EEG	LR, SVM, NB		Multimodal
[57]	Metal stress	EEG	LR, SVM, NB	85.71%	Unimodal
[71]	Construction worker stress	EEG	*k*-NN, GDA, SVM	*k*-NN - 65.80%, QSVM-69.62%, GSVM-80.32%	Unimodal

### Emotion/stress detection using EEG

EEG is a noninvasive way to measure electrical responses generated by the outer layers of the cortex, primarily pyramidal cells. It has been used to investigate neural activity during arousal, stress, depression, anxiety or various other emotions. Several studies have applied ML methods to classify and/or predict emotional brain states based on EEG activity [72, 73]. For example, Chen et al. [74] designed a neural feedback system to predict and classify anxiety states using EEG signals during the resting state from 34 subjects. Anxiety was calculated using power spectral density (PSD), and then SVM was used to classify anxious and non-anxious states. Shon et al. [67] integrated genetic algorithm (GA)-based features in the ML pipeline along with a *k*-NN classifier to detect stress in EEG signals. The model was evaluated using DEAP data set [56] for the identification of emotional stress state. Other work also used the publicly available DEAP data set for emotion recognition in virtual environments [68]. Based on Russell’s circumplex model, statistical features, high order crossing (HOC) features and powerbands were extracted from the EEG signals, and affective state classification was performed using SVM and RF. In major depressive disorder (MDD, *n* = 32), Duan et al. [69] extracted interhemispheric asymmetry and cross-correlation features from EEG signals and combined these in a classification using *k*-NN, SVM and convolutional neural networks (CNN). Similarly, in other research by Omar [70], frontal lobe EEG data were used to identify stressed patients. Fast Fourier transformation (FFT) was applied to extract features from the signal, which were then passed to ML models, such as SVM and NB, for subject-wise classification of control and stress groups. Table 1 shows a summary of ML models used for arousal detection and their performance.

### Machine learning and VRET

**Fig. 1:**
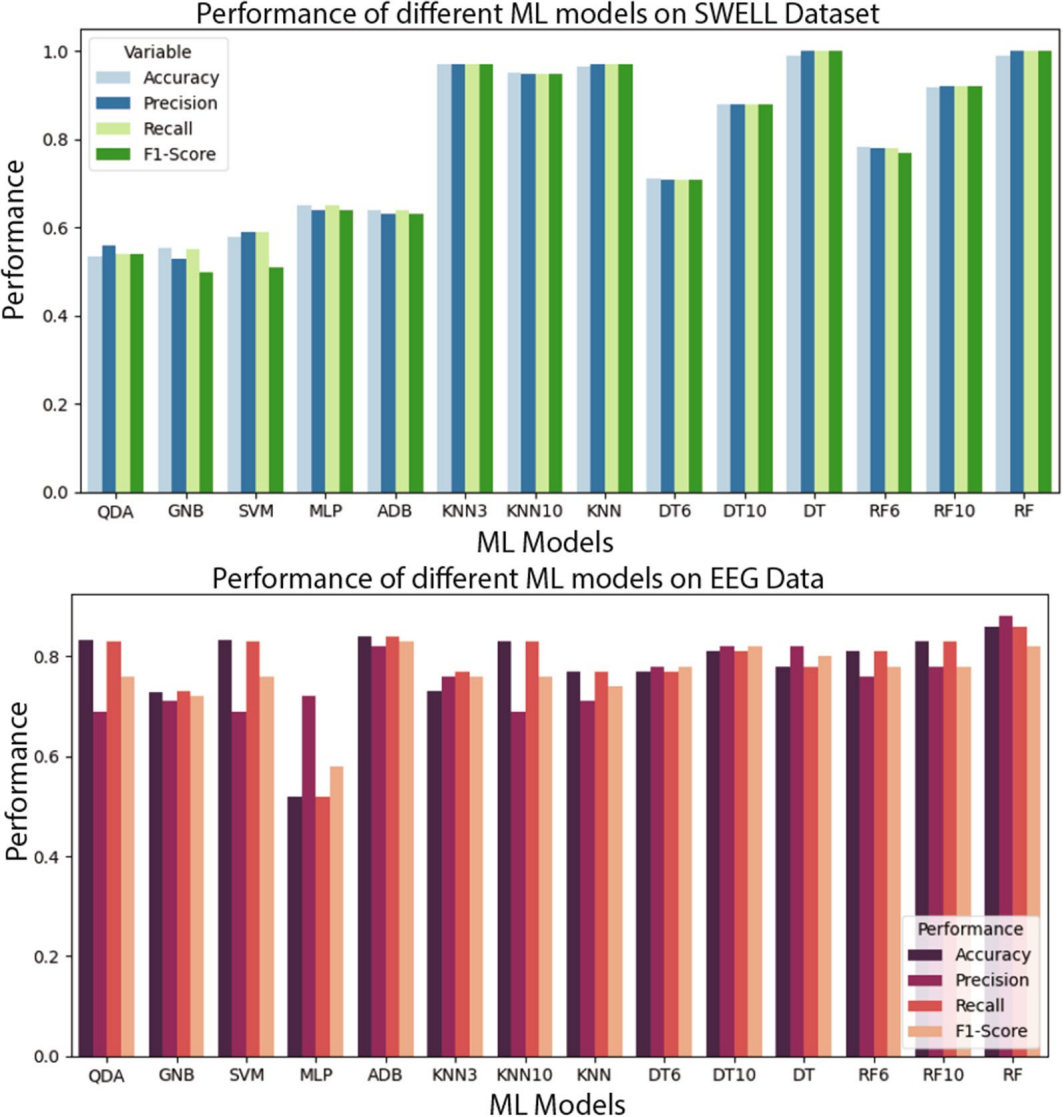
Figures show the performance (accuracy, precision, recall and *F*1-Score) of the publicly available data set that we used to train our model. Here, we consider QDA, GNB, SVM, MLP, ADB, KNN, DT and RF ML models. KNN, DT and RF have been used with multiple parameter settings. The figure on the top shows the performance of the SWELL [80] data set and the figure on the bottom shows the performance on the EEG data set of [79]

Balan et al. [3] used the publicly available DEAP [56] database and applied various ML algorithms for classifying the six basic emotions joy, anger, sadness, disgust, surprise and fear, based on the physiological data. They presented the stages of model development and its evaluation in a virtual environment with gradual stimulus exposure for acrophobia treatment, accompanied by physiological signals monitoring. In [62], authors used a hybrid ML technique using *k*-Means++ clustering algorithm and principal component analysis (PCA) to cluster drug addicts to find out the relationship between cardiac physiological characteristic data and treatment effect. The author showed the relationship between cardiac physiological characteristics and treatment effects using virtual reality. Other research [64] used a single session VRET for patients with spider phobia, including clinical, neuroimaging (functional magnetic resonance imaging, fMRI), and genetic data for baseline and post-treatment (after 6 months) analysis. They claimed a 30% reduction in spider phobia, assessed psychometrically, and a 50% reduction in individual distance avoidance tests using behavioural patterns. From these literature reviews, we systematically picked the widely used ML algorithms to develop our ML pipeline. In Fig. 1, we showed the the performance (accuracy, precision, recall and *F*1-Score) of the publicly available data set that we used to train our model. based on our careful existing literature review we considered Gaussian Naïve Bayes (GNB), quadratic discriminant analysis (QDA), support vector machine (SVM), multilayer perceptron (MLP), AdaBoost (ADB), *k*-nearhood neighbour (KNN), decision tree (DT) and random forest (RF) ML models with multiple parameter settings.

## ML model pipeline and data set

**Fig. 2:**
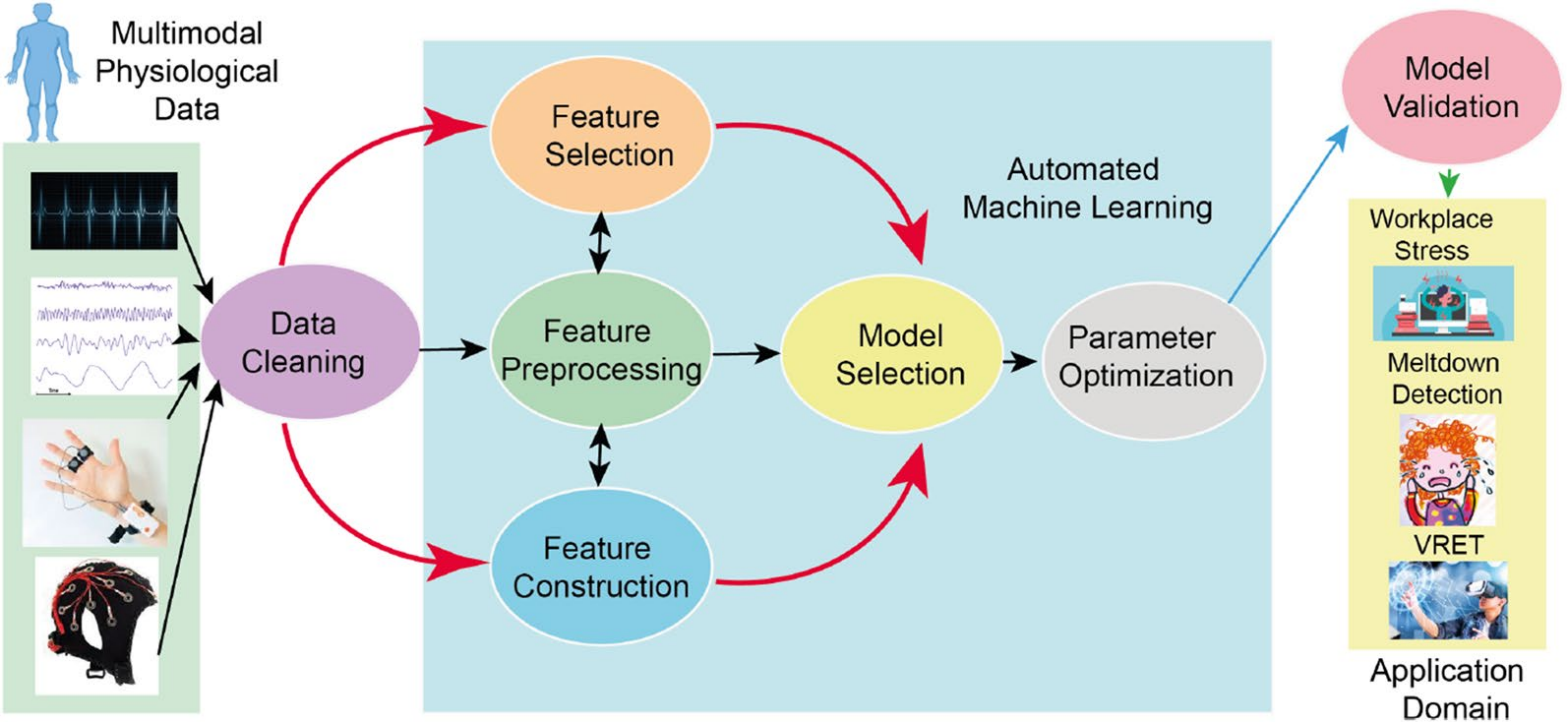
Proposed machine learning pipeline: we collect EEG and multimodal physiological data from suitable sensors. To clean the data for further processing, we used individual phases of feature selection, feature prepossessing and feature constructions for model selection which was used for parameter optimisation. This process was repeated using automated ML for the best possible outcome from the collected data set. After model validation, we use our trained model for meltdown moment detection, workplace stress detection, VRET and/or other domains where arousal detection is crucial

First, we collected EEG and multimodal physiological data from suitable sensors. Then we cleaned the data for further processing. Here we used individual phases of feature selection, feature prepossessing and feature constructions for model selection used for parameter optimisation. This process was repeated using automated ML for the best possible outcome from the collected data set. After the model validation, we apply our trained model to VRET and/or other domains where arousal detection is crucial. Figure 2 shows the proposed ML pipeline.

### Feature extraction for real-time data analysis

Different feature for real-time data analysis has been extracted form [71, 75–77]. In the domain of ML selection of useful features from data to identify stress levels is crucial. A better selection of features can improve the efficacy of the classification algorithm with a reduced computational cost. For the case of EEG signals, we can consider a large number of features both in frequency domains and in time. However, learning the possible combination of subsets and comparing their performance requires extra computational burden.

**Fig. 3:**
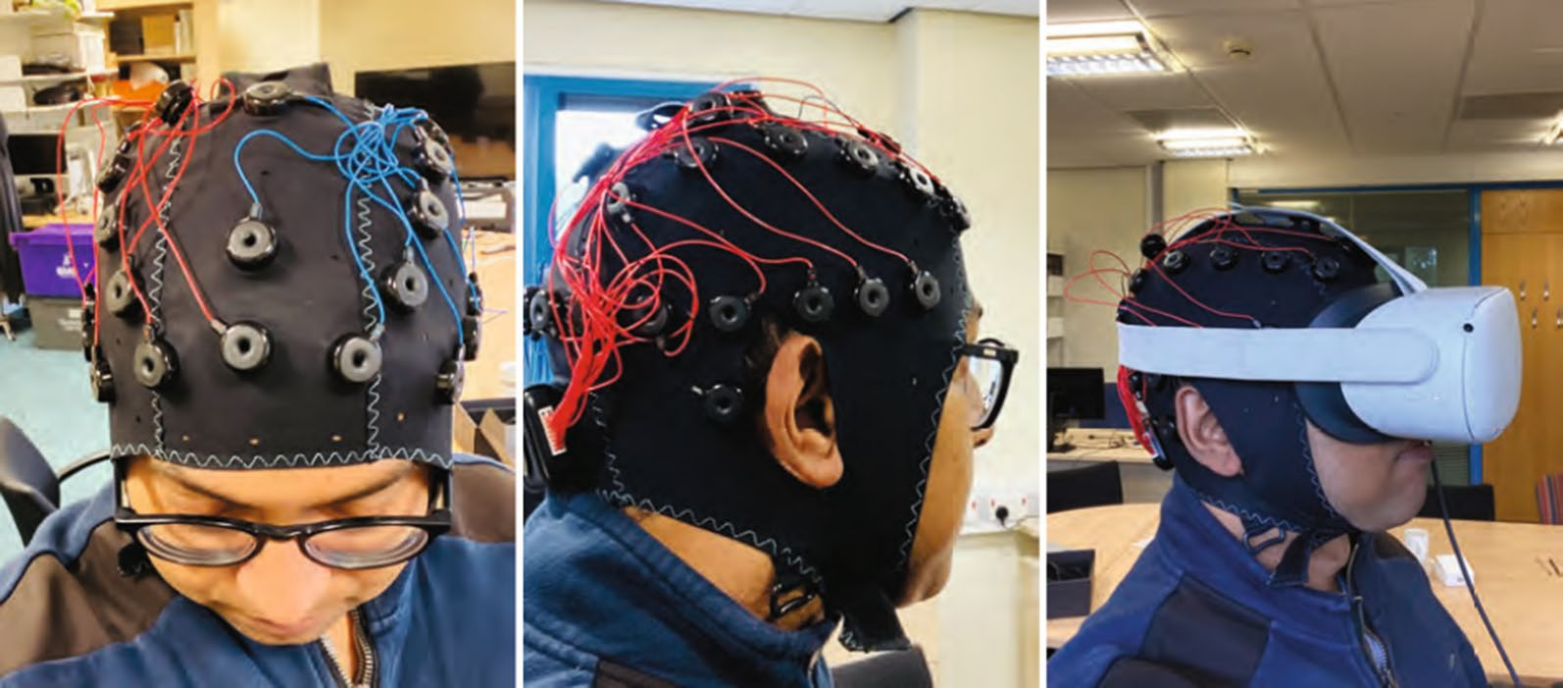
Data acquisition using emotive EPOC flex. The figure on the left shows the top view, the figure in the middle shows a side view of emotive EPOC flex, and the figure on the right shows the data acquisition phase using emotive EPOC flex and Oculus Quest 2 head-mounted displays

If we record our EEG signal with 128 Hz, calculating any feature over one single EEG reading is not informative enough, as 128 data points per second will be massive. This issue can be overcome by introducing the concept of a window, which is a continuous block of readings. Different studies claimed that a window size between 3 to 12 s is an adequate window size while classifying mental status using EEG signals. A sliding window approach is another alternative. However, research shows that with an added cost of computation burden. Here in our experiment, we have used a fixed size window of 5 s with 128 Hz of sampling frequency. Figure 3 shows data acquisition using emotive EPOC flex. The figure on the left shows the top view, the figure in the middle shows a side view of emotive EPOC flex, and the figure on the right shows the data acquisition phase using emotive EPOC flex and Oculus Quest 2 head-mounted displays.

The mean of the raw signal [75]:

1 $$\begin{aligned} \mu _X = \frac{1}{N}\sum _{n=1}^{N}X\left( n \right) , \end{aligned}$$

where $X\left( n \right)$ represents the value of the $n{\mathrm{th}}$ sample of the raw EEG signal, $n = 1,\ldots N.$ The standard deviation of the raw signal:

2 $$\begin{aligned} \sigma _X = \sqrt{\frac{1}{N-1}\sum _{n=1}^{N}\left( X\left( n \right) -\mu _X\right) ^2}. \end{aligned}$$

The mean of the absolute values of the first differences of the raw signal:

3 $$\begin{aligned} \delta _X = \frac{1}{N-1}\sum _{n=1}^{N-1}\left| X\left( n+1 \right) -X\left( n \right) \right| . \end{aligned}$$

The mean of the absolute values of the second differences of the raw signal:

4 $$\begin{aligned} \gamma _X = \frac{1}{N-2}\sum _{n=1}^{N-2}\left| X\left( n+2 \right) -X\left( n \right) \right| . \end{aligned}$$

The means of the absolute values of the first differences of the normalised signals:

5 $$\begin{aligned} \tilde{\delta }_X = \frac{1}{N}\sum _{n=1}^{N-1}\left| \tilde{X}\left( n+1 \right) -\tilde{X}\left( n \right) \right| = \frac{\delta _X}{\sigma _X}, \end{aligned}$$

where $\tilde{X}\left( n \right) = \frac{X\left( n \right) -\mu _X}{\sigma _X}$, ${\mu _X}$ and ${\sigma _X}$ are the means and standard deviations of *X*.

The means of the absolute values of the second difference of the normalised signals:

6 $$\begin{aligned} \tilde{\gamma }_X = \sum _{n=1}^{N-2}\left| \tilde{X}\left( n+2 \right) -\tilde{X}\left( n \right) \right| = \frac{\gamma _X}{\sigma _X}. \end{aligned}$$

Time and frequency domain features, extracted from EEG signals.

The maximum amplitude of channel *j* up to sample *i* (cumulative maximum):

7 $$\begin{aligned} \text{Cmax}_{ij} = \text{max}\left(\text{EEG}_{1:i, j}\right) . \end{aligned}$$

Minimum amplitude of channel *j* up to sample *i* (cumulative minimum):

8 $$\begin{aligned} \text{Cmin}_{ij} = \text{min}\left(\text{EEG}_{1:i, j}\right) . \end{aligned}$$

The average absolute value of amplitude among different channels (mean value):

9 $$\begin{aligned} \text{MAV}_{j} = \frac{\sum _{i=1}^{N} \text{EEG}_{i,j}}{N}. \end{aligned}$$

Median of the signal among different EEG channels (median value):

10 $$\begin{aligned} \text{Med}_{j} = \text{sort}(\text{EEG})_{\frac{N+1}{2},j}. \end{aligned}$$

Minimum amplitude among different channels (smallest window elements):

11 $$\begin{aligned} \text{Min}_j = \underset{i}{\text{min}}\ \text{EEG}_{ij}. \end{aligned}$$

Median of the signal of channel *j* in a window with size *k* samples (moving median with window size *k*):

12 $$\begin{aligned} \text{MovMed}_{i,j}=\text{Median}\left(\text{EEG}_{i:i+k-1,j}\right) . \end{aligned}$$

Difference between maximum and minimum of the EEG signals amplitude among different EEG channels (maximum-to-minimum difference):

13 $$\begin{aligned} \text{MaxMin}_j = \underset{i}{\text{max}}\ \text{EEG}_{ij}-\underset{i}{\text{min}}\ \text{EEG}_{ij}. \end{aligned}$$

Norm 2 of the EEG signals divided by the square root of the number of samples among different EEG channels (root-mean-square level):

14 $$\begin{aligned} \text{RMS}_j=\sqrt{\frac{\sum _{i=1}^{N} \text{EEG}_{i,j}^2}{N}}. \end{aligned}$$

Maximum of the EEG signal amplitude divided by the $\text{RMS}_j$ (peak-magnitude-to-RMS ratio):

15 $$\begin{aligned} \text{PRMS}_j=\frac{\left| \text{EEG}_{:,j}\right| _{\infty }}{\sqrt{\frac{\sum _{i=1}^{N} \text{EEG}_{i,j}^2}{N}}}. \end{aligned}$$

Norm of the EEG signals among different channels in each window (root-sum-of-squares level):

16 $$\begin{aligned} \text{RSS}_j=\sqrt{\sum _{i=1}^{N} \left| \text{EEG}_{i,j}\right| ^2}. \end{aligned}$$

Deviation of EEG signals among different channels in each window (standard deviation):

17 $$\begin{aligned} \text{STD}_j=\sqrt{\frac{1}{N-1}\sum _{i=1}^{N} \text{EEG}_{i,j}^2}. \end{aligned}$$

The variance of the signal EEG amplitude among different channels (variance):

18 $$\begin{aligned} \text{VAR}_j=\frac{1}{N-1}\sum _{i=1}^{N} \text{EEG}_{i,j}^2. \end{aligned}$$

The maximum value of EEG amplitude among different channels in the time domain (peak):

19 $$\begin{aligned} \text{Pk}_j = \underset{i}{\text{max}}\ \text{EEG}_{ij}. \end{aligned}$$

Location of maximum EEG amplitude among channels (peak location):

20 $$\begin{aligned} \text{LPk}_j = \underset{i}{\text{argmax}}\ \text{EEG}_{ij}. \end{aligned}$$

The time between EEG signal peaks between the various windows (peak to peak):

21 $$\begin{aligned} \text{PP}_j = \text{LPk}_j - \underset{i,i \ne \text{LPk}_j}{\text{argmax}}\ \text{EEG}_{ij}. \end{aligned}$$

Shows the sharpness of EEG signals peak (kurtosis):

22 $$\begin{aligned} k_j=\frac{\frac{1}{N}\sum _i\left(\text{EEG}_{ij}-\text{MAV}_j\right) ^4}{\left( \frac{1}{N}\sum _i\left(\text{EEG}_{ij}-\text{MAV}_j\right) ^2\right) ^2}. \end{aligned}$$

Power of the EEG signal in channel *j* in the frequency domain in the interval [8 Hz, 15 Hz] (Alpha mean power):

23 $$\begin{aligned} \alpha _j=\text{power}\left(\text{EEG}_{:, j},f\in \left[8\,\text{Hz},\,15\,\text{Hz}\right] \right) . \end{aligned}$$

Power of the signal in Beta interval (Beta mean power):

24 $$\begin{aligned} \beta _j=\text{power}\left(\text{EEG}_{:, j},f\in \left[16\,\text{Hz}, 31\,\text{Hz}\right] \right) . \end{aligned}$$

Power of the signal in Delta interval (Delta mean power):

25 $$\begin{aligned} \delta _j=\text{power}\left(\text{EEG}_{:, j},f\in \left[0\,\text{Hz},\, 4\text{Hz}\right] \right) . \end{aligned}$$

Power of the signal in Theta interval (Theta mean power):

26 $$\begin{aligned} \theta _j=\text{power}\left(\text{EEG}_{:, j},f\in \left[4\,\text{Hz},\,7\,\text{Hz}\right] \right) . \end{aligned}$$

Level of happiness (valence) [71]:

27 $$\begin{aligned} V= \frac{\alpha \left( F4\right) }{\beta \left( F4\right) } - \frac{\alpha \left( F3\right) }{\beta \left( F3\right) }. \end{aligned}$$

We have used the sampling frequency of the signal to 128 Hz. If we want to calculate the features on one individual EEG reading then may not be much informative, due to a large number of data points. To overcome this problem, we have used blocks of continuous readings which are also termed windows. We extracted our features from these windows. Previous studies show that the window size between 3 to 12 s is an effective window length while classifying the mental status from EEG signals [71].

Level of excitement (arousal) [71]:

28 $$\begin{aligned} A= \frac{\alpha \left( F3+F4+AF3+AF4\right) }{\beta \left( F3+F4+AF3+AF4\right) }. \end{aligned}$$

Half of the signal power of channel *j* is distributed in the frequencies less than $\text{MEDF}_j$ (median frequency):

29 $$\begin{aligned} \text{power}\left(\text{EEG}_{:, j},f\in \left[0\,\text{Hz},\,\text{MEDF}_j\right] \right) = \text{power}\left(\text{EEG}_{:, j},f\in \left[ \text{MEDF}_j,64\,\text{Hz}\right] \right) . \end{aligned}$$

If arousal is less than 4 and valence is between 4 and 6, as in the following equation, it is defined as calm [77]:

30 $$\begin{aligned} \left( \text{arousal}< 4\right) \cap (4< \text{valence} < 6), \end{aligned}$$

where arousal stands for a range from calm to excited, while valence presents a range from unpleasant to pleasant. If arousal exceeds 5 and valence is less than 3, as in the following equation, it is defined as a stress state [77]:

31 $$\begin{aligned} \left( \text{arousal} > 5\right) \cap ( \text{valence} < 3). \end{aligned}$$

The frequency range are [78]:

- $$\delta : 0.5-4 \, \text{hertz};$$
- $$\theta : 4-8 \, \text{hertz};$$
- $$\alpha : 8-12 \, \text{hertz};$$
- $$\beta : 12-30 \, \text{hertz};$$
- $$\gamma : > 30 \, \text{hertz}.$$

### Data set

In the first stage, we explored three publicly available data sets. The first one is the SWELL data set of [80]. The authors calculated the inter-beat interval (IBI) between peaks in electrocardiographic (ECG) signals. Then, the heart rate variability (HRV) index was computed on a 5 min IBI array by appending the new IBI sample to the array in a repeated manner. The data set was manually annotated with the conditions under which the data were collected. This data set has 204,885 samples with 75 features and 3 labelled classes. Here, 25 people performed regular cognitive activities, including reading e-mails, writing reports, searching, and making presentations under manipulated working conditions. We used a second publicly available data set of [81], which was initially inspired from [82], with HRV data to train our proposed ML model and determine arousal levels.

We also used a third publicly available data set titled ‘EEG during Mental Arithmetic Task Performance’ [79] to explore EEG recordings of 36 participants during resting state and while doing an arithmetic task. The dataset was collected using a Neurocom monopolar EEG 23-channel system device. Electrodes (Fp1, Fp2, F3, F4, Fz, F7, F8, C3, C4, Cz, P3, P4, Pz, O1, O2, T3, T4, T5, T6) were placed on the scalp using international 10/20 standard. The sampling rate for each channel was 500 Hz with a high-pass filter of 0.5 Hz and a low-pass filter of 45 Hz cut-off frequency. In the experimental manipulation, participants were asked to solve mental arithmetic questions to increase cognitive load and induce stress, thus, evoking higher arousal states.

## Result analysis

**Fig. 4:**
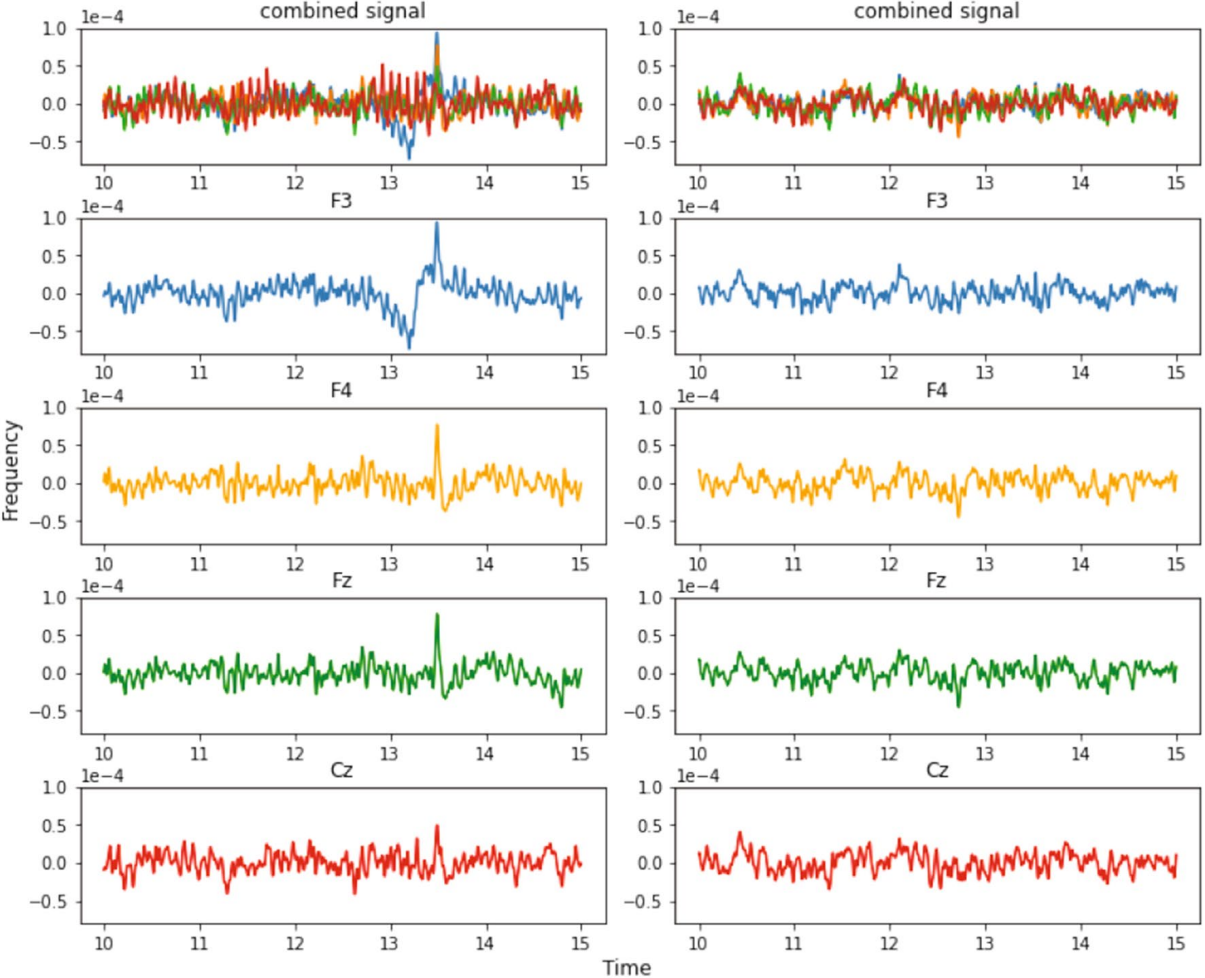
The time domain representation of EEG data of [79]. The top figures show the combined representations. Figures on the left show the initial condition and figures on the right show the stressed condition in channels F3, F4, Fz, and Cz. We can clearly see the increase of oscillatory patterns of the signal from initial to stressful conditions

In this study, we took the dataset of EEG signals during mental arithmetic tasks^1^ [79]. Decomposed EEG signals for a duration of 5 s before and during an arithmetic task are shown in Fig. 4. The signals were in edf format, which is converted to epochs and their statistical features (mean, std, ptp, var, minim, maxim, argminim, argmaxim, skewness and kurtosis) were calculated. These were then used for the classification of the signals. RF model was used for this purpose which gave an accuracy of 87.5%.

Figure 4 shows the time-domain representation of EEG signal of [79]. In this figure, plots on the left show recordings during the initial condition and plots on the right during the stressed condition in channels F3, F4, Fz, and Cz. We can clearly see the increase of oscillatory patterns of the signal from initial to stressful conditions.

**Fig. 5:**
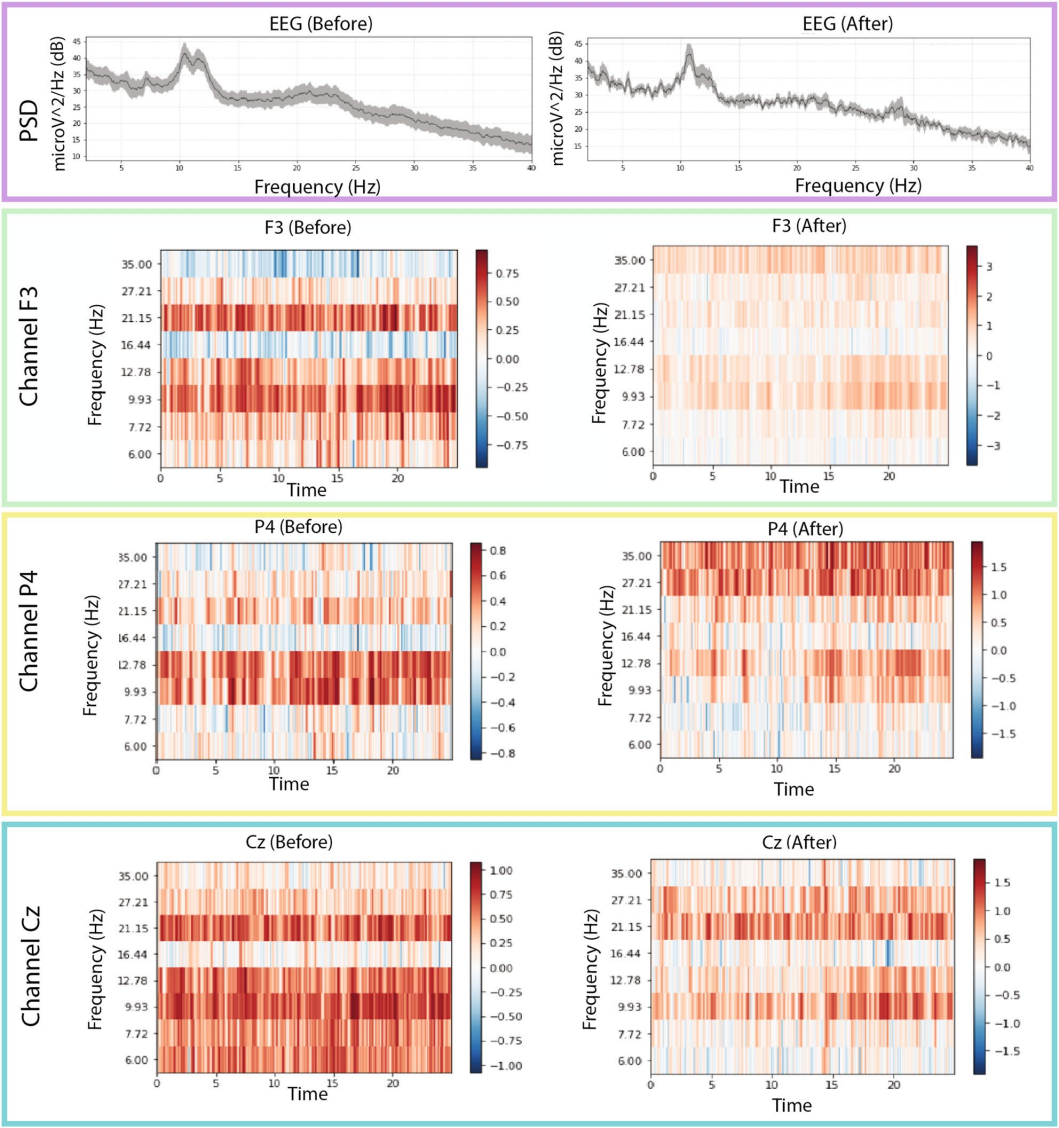
Average frequency content of signal before and during the arithmetic task using [79] data set. We can clearly see changes in excitation levels. The figure on the left shows the initial level, whereas the right figure shows the stressed condition during mathematical problem-solving. The figures were generated using the open source python package MNE-Python [83]

**Fig. 6:**
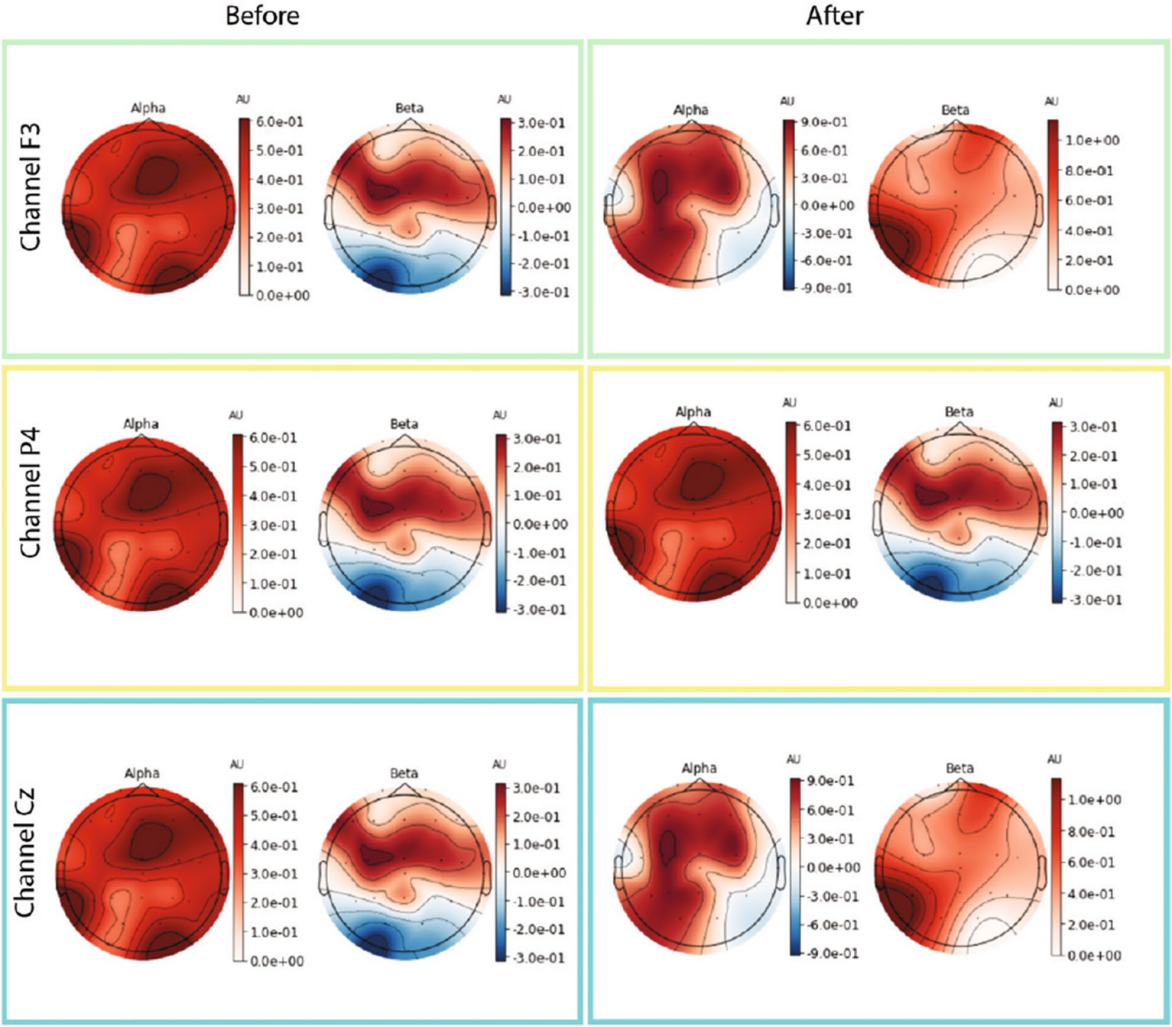
Images above show the time–frequency representations plotted using power plot topographic maps. Changes in power spectral density can be seen for individual channels before and during the stressed conditions. The figures were generated using the open source python package MNE-Python [83]

**Fig. 7:**
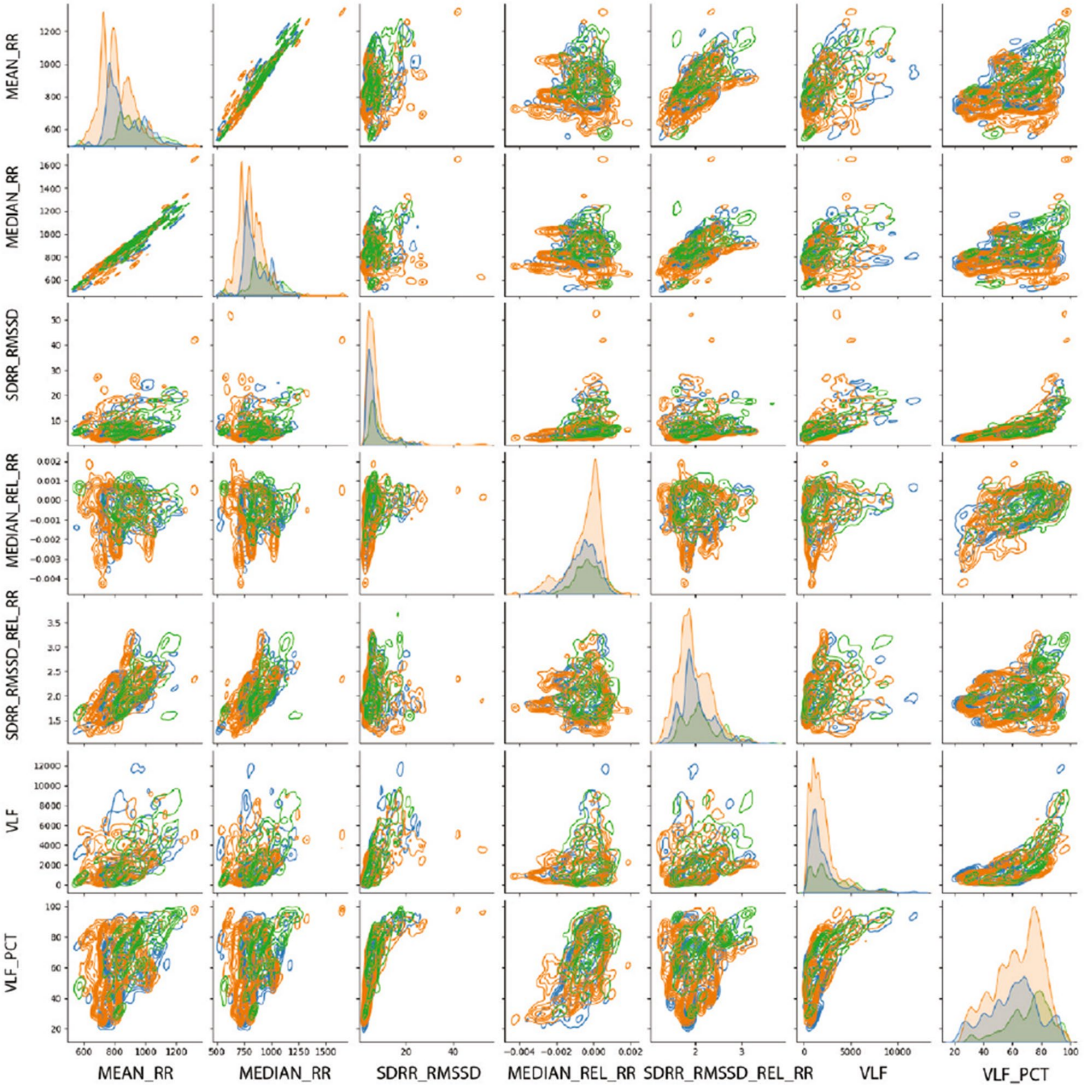
The figure shows the pairplot of a few notable features MEAN-RR, MEDIAN-RR, SDRR-RMSSD, MEDIAN-REL-RR, SDRR-RMSSD-REL-RR, VLF, VLF-PCT from SWELL dataset [80]. These statistical features have been used for the classification of the signals aiming at arousal detection. This publicly available HRV dataset has been used to train our ML models

**Fig. 8:**
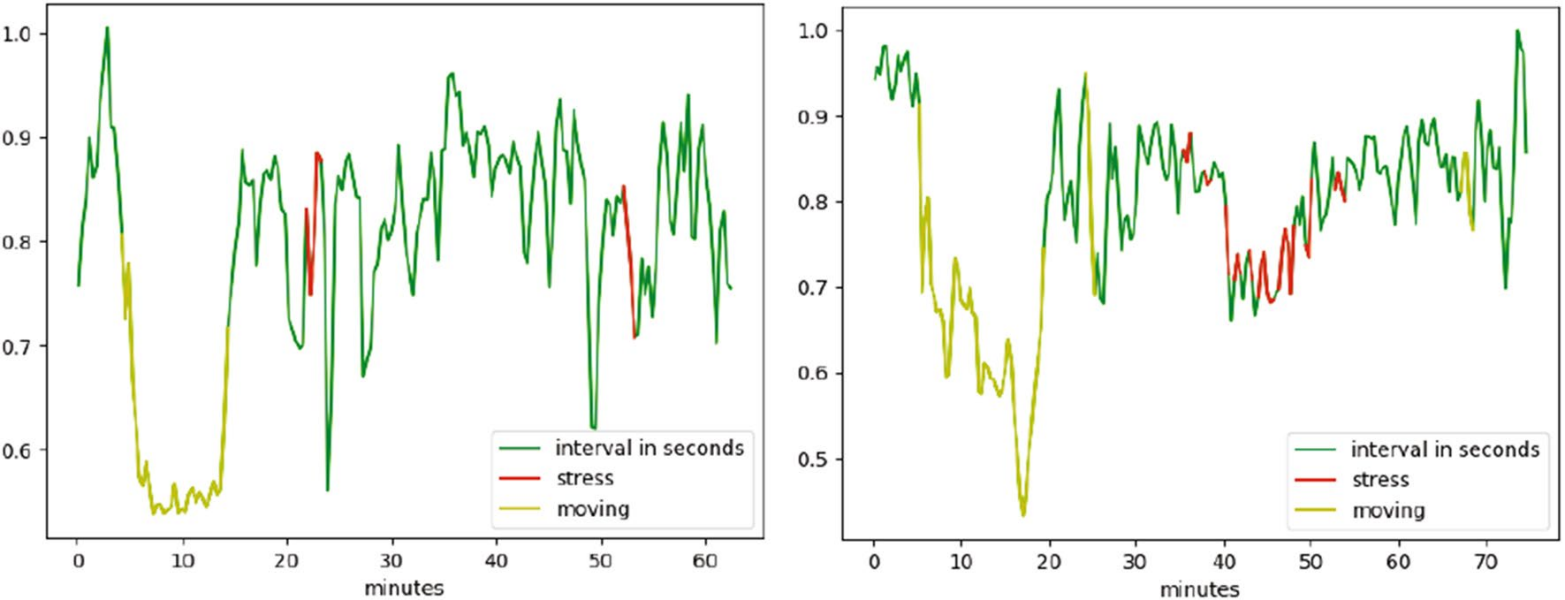
The figure shows the prediction of stressful moments from the HRV data set generated by [81] inspired from [82]. We used the publicly available data set of [81] to train our proposed ML model for VRET and determine momentary stress states

**Fig. 9:**
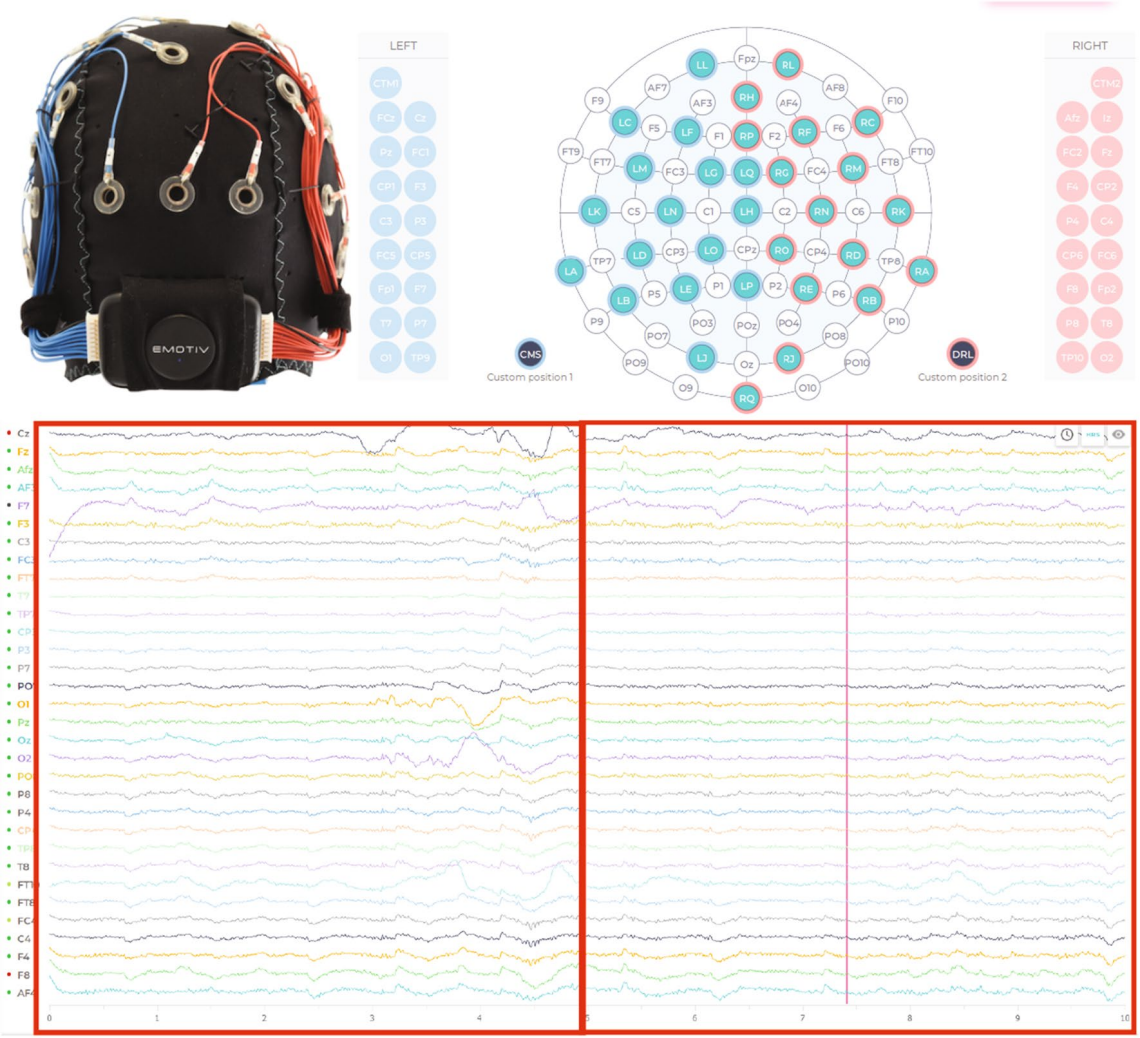
Figure on the top-left show an emotive EPOC flex while the top-right is showing its 10–20 diagram. The bottom one shows a sample signal acquisition with its different electrodes. The red rectangular box is showing a window of 5 s from where data were collected with a 128-Hz sampling frequency

Figure 5 shows average frequency content of signal epochs before and during solving arithmetic tasks using [79] data set. We can see some changes in excitation levels. The figures on the left show the signal in a relaxed state, whereas the figures on the right depict the signals under stress while performing mental arithmetic tasks. Similarly, subsequent images in Fig. 5 show the time–frequency analysis of individual channels (F3, Cz, and P4) generated using power plots and topographic maps. A significant difference can be seen between plots before and during evoked stress states (Fig. 6). Figure 7 shows the pair plot of a few notable features MEAN-RR, MEDIAN-RR, SDRR-RMSSD, MEDIAN-REL-RR, SDRR-RMSSD-REL-RR, VLF, VLF-PCT from SWELL dataset [80]. These statistical features have been used to classify the signals aiming for arousal detection. This publicly available HRV dataset has been used to train our ML models. Figure 8 shows the prediction of stressful moments from the HRV data set generated by [81] inspired from [82]. We used the publicly available data set of [81] to train our proposed ML model and determine momentary stressful states. Figure 9 shows the performance (accuracy, precision, recall and *F*1-Score) of the publicly available data set that we have used to train our model. Here we consider Gaussian Naïve Bayes (GNB), quadratic discriminant analysis (QDA), support vector machine (SVM), multilayer perceptron (MLP), AdaBoost (ADB), *k*-nearhood neighbour (KNN), decision tree (DT) and random forest (RF) ML models. KNN, DT and RF have been used with multiple parameter settings. The figure on the top shows the performance of the SWELL [80] data set and the figure on the bottom shows the performance on the EEG data set of [79].

## Biofeedback for VRET

**Fig. 10:**
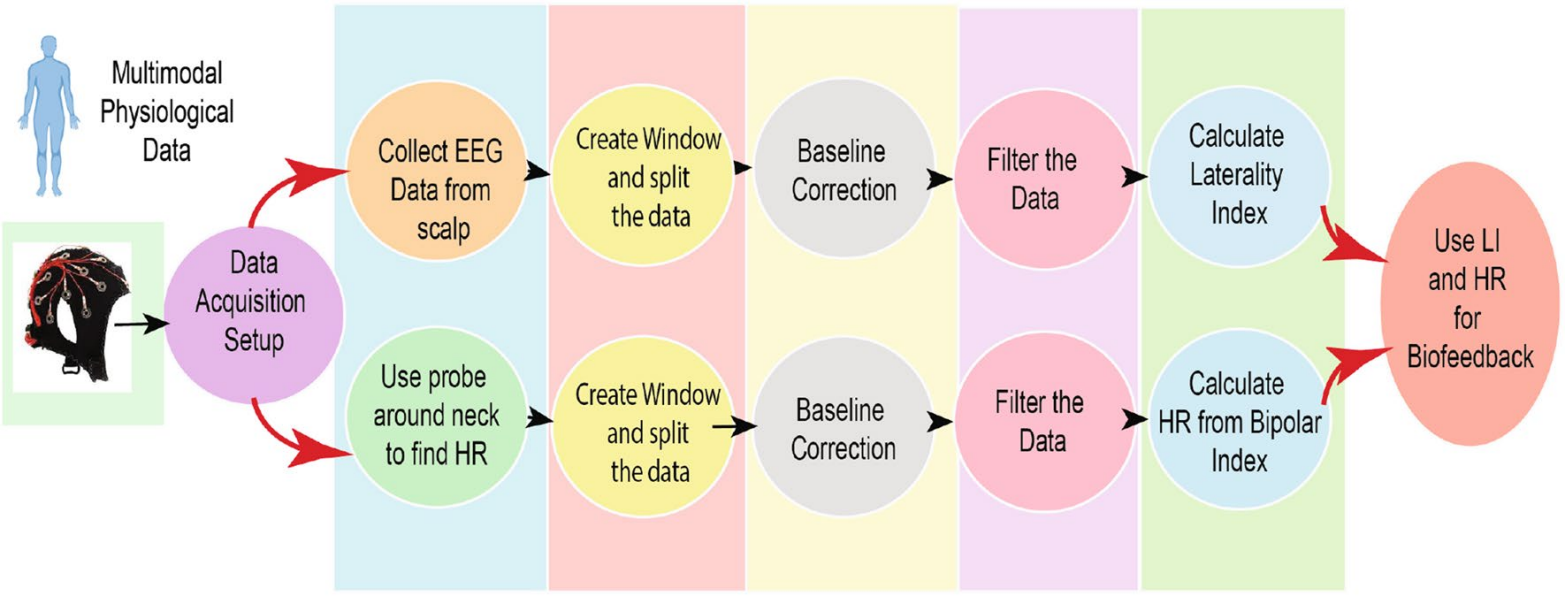
Biofeedback for VRET: to reduce the interference, we had to target to minimise the use of the number of sensors. We planned to use heart rate, so it was challenging to calculate heart rate using emotive EPOC flex. We used electrodes FT9 and FT10 to determine our heart rate. We placed the probe across the neck. We first performed the baseline correction for the acquired raw signal and then filtered the data. Afterwards, we calculated the bipolar difference to determine the heart rate. On the other side, we used 5 s window for our EEG data acquisition. Then we systematically did the baseline correction, filtered the data and used electrodes *F*3, *F*4, *AF*3 and *AF*4 to calculate the literality index. Then we used calculated heart rate and literality index as forms of biofeedback

**Fig. 11:**
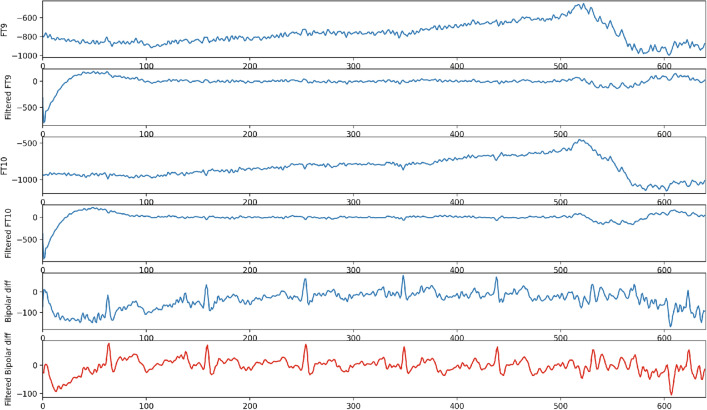
Heart rate extraction from EEG data. We used electrodes FT9 and FT10 to determine our heart rate. We placed the probe across the neck. For the acquired raw signal first, we performed the baseline correction and then filtered the data. Afterwards, we calculated the bipolar difference to determine the heart rate

**Fig. 12:**
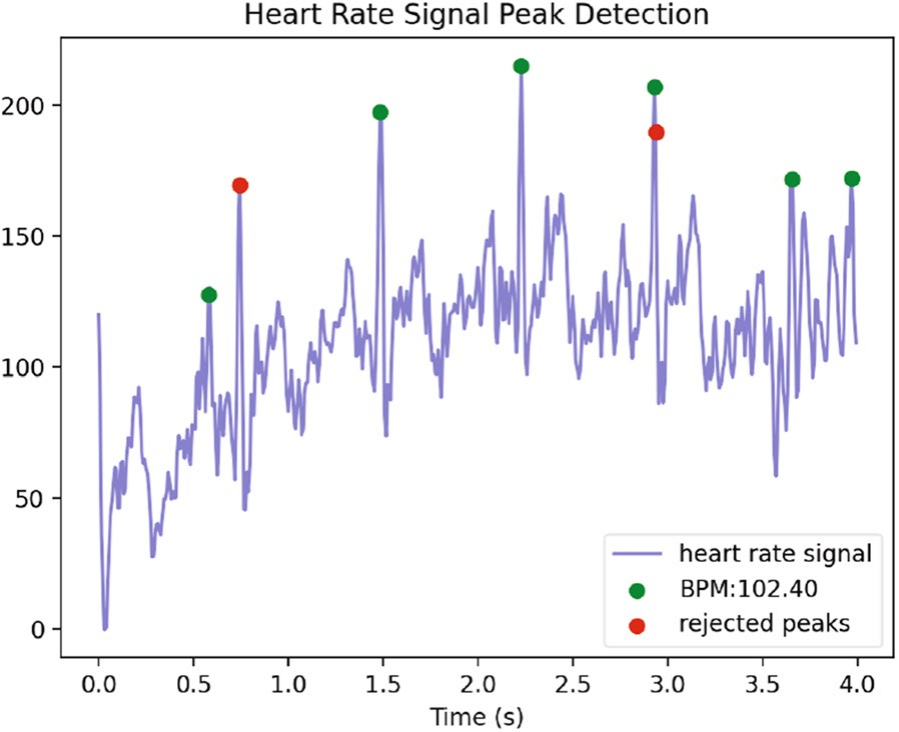
Heart rate calculation from EEG data. Here, we have determined the peaks to calculate the heart rate where we had to reject the false peaks systematically

**Fig. 13:**
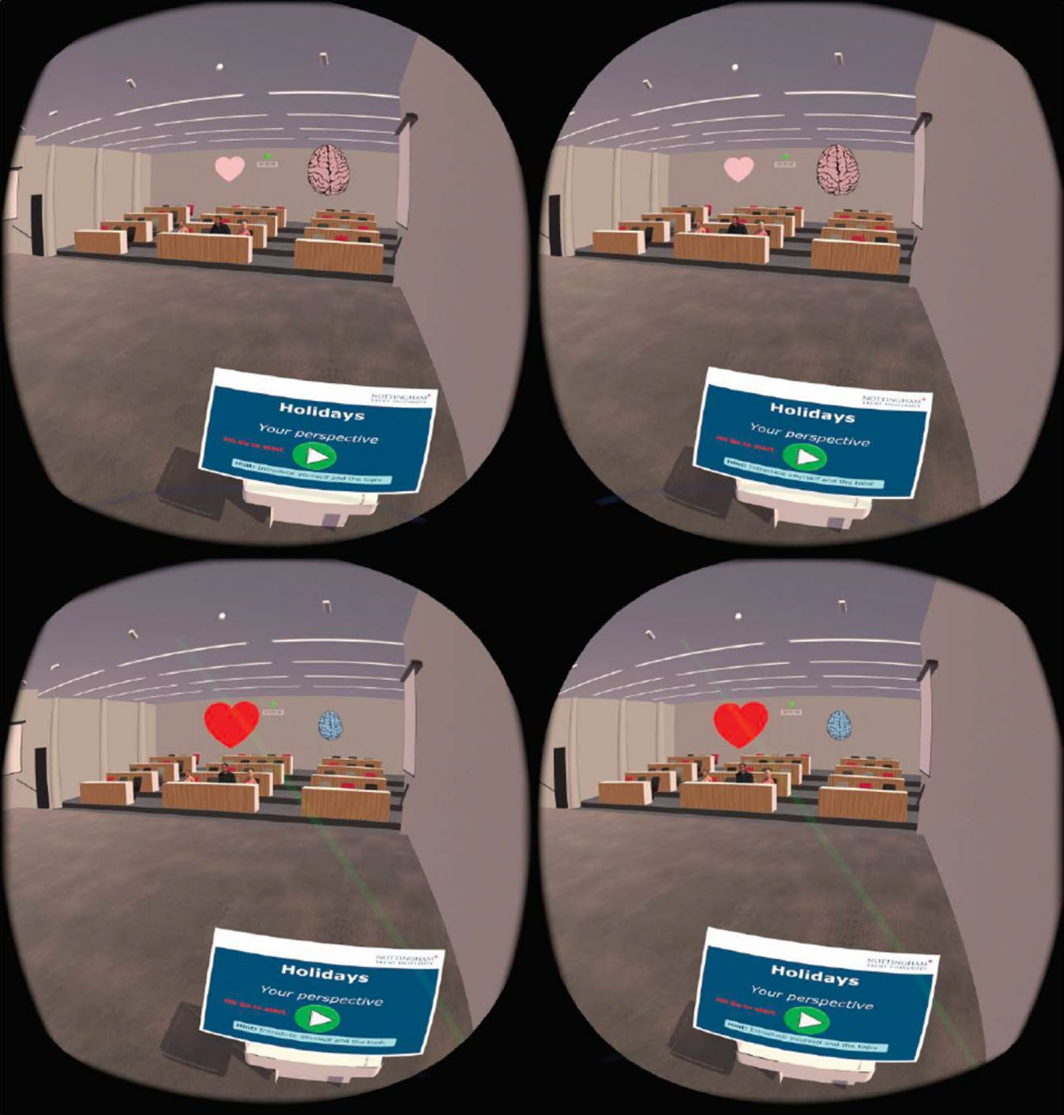
Snapshot of some virtual environment where biofeedback has been used. In the environment, we can see the image of the heart and brain with different colours and shapes. The size and the colours of the heart and brain were mapped with the level of arousal. A small pink heart represents a normal condition. However, as the heart rate increases, its colour and size also change in the VR environment. The colour and size of the brain are related to the laterality index

As the Related work Sect. 2 indicates, the state-of-the-art development clearly lacks one key direction; there is no attempt at real-time biofeedback for VRET intervention. Here in this research, a vital part of our development of VRET is the integration of bio-signals, such as heart rate, heart rate variability or cortical arousal, to assess and ameliorate physiological distress states (e.g., fear or anxiety-induced arousal) during exposure. We have created a VR environment and a mechanism to provide biofeedback during the VRET session. We acquired the cortical arousal using an emotive EPOC flex. After a near real-time processing of the EEG signals (as we considered a window approach, there was a constant delay equivalent to window length as shown in Fig. 9 plus an insignificant variable delay for signal processing time). To reduce the interference, we had to target to minimise the use of the number of sensors. We planned to use heart rate, so it was challenging to calculate heart rate using emotive EPOC flex. In the Fig. 9, we can see an emotive EPOC flex with its adjustable 10–20 diagram. The bottom segment shows a sample signal collected using its different electrodes. The red rectangular box shows a window of 5 s from where data were collected with a 128-Hz sampling frequency. We used electrodes FT9 and FT10 to determine our heart rate. We placed the probe across the neck. For the acquired raw signal, first, we performed the baseline correction and then filtered the data. Afterwards, we calculated the bipolar difference to determine the heart rate. On the other side, we used 5-s window for our EEG data acquisition. Then we systematically did the baseline correction, filtered the data and used electrodes *F*3, *F*4, *AF*3 and *AF*4 to calculate the literality index. Then we used calculated heart rate and literality index as forms of biofeedback. Figure 10 shows the block diagram of the feedback generation process. During the heart rate calculation from EEG data, we used electrodes FT9 and FT10 to determine our heart rate. We placed the probe across the neck. For the acquired raw signal first, we performed the baseline correction and then filtered the data. Afterwards, we calculated the bipolar difference to determine the heart rate. Figure 11 shows the time-domain representation of the signals at their different stages of processing. From Fig. 12, we have determined the peaks to calculate the heart rate where we had to reject the false one systematically. Figure 13 shows a few snapshots of the virtual environment where biofeedback has been used. In the environment, we can see the image of the heart and brain with different colours and shapes. The size and the colours of the heart and brain were mapped with the level of arousal. A small pink heart represents a normal condition. However, as the heart rate increases, its colour and size also change in the VR environment. The colour and size of the brain are related to the laterality index.

We believe we have invented the wheel here, and there was no previous wheel to compare. Biofeedback-based intervention for VRET is a novel invention. Earlier, there was no existing literature or published work of biofeedback for VRET to compare our results. We have a future plan to recreate the experiment with and without biofeedback and compare the results. We also have the plan to deploy our proposed machine-learning framework for VRET with biofeedback and compare the results. Yet, we have to keep it in mind that for the same ML algorithm with a fixed parameter settings, if we use a different set of data then the results may vary slightly as showed by [48].

## Challenges and future research directions

As we mentioned in the Related work section (Sect. 2), this work is derived through multi-disciplinary research. So, diverse open challenges have been identified. Some of the key issues are:

- The real-time analysis of the ML data. Stream processing will be one of the next challenges that we want to overcome for the same problem.
- One VRET session for a specific kind of anxiety might be very different from another VRET session with a different kind of anxiety or disorder. For a validation check, a comparison of a development with a new idea and its implementation to an existing work might be very challenging.
- The placement of the BCI electrodes is an important consideration, and interesting to investigate further to determine the most relevant regions of the brain to monitor arousal.
- To provide biofeedback for the VRET, haptic feedback could be used. It is yet to explore how real-time biofeedback can be provided. We need to investigate that at incorporate.
- In future, additional sensor/polar devices, chest straps and/or wristbands could be used to collect further types of signals. Moreover, additional data should be collected from different experimental conditions to further improve efficacy.

## Conclusion

In self-guided VRET, participants can gradually increase their exposure to anxiety-evoking stimuli (like audience size, audience reaction, the salience of self, etc.) to desensitise and reduce momentary anxiety and arousal states, facilitating amelioration of PSA over time. However, creating this VR environment and determining anxiety-induced arousal or momentary stress states is an open challenge. In this work, we showed which selection of parameters and ML models can facilitate arousal detection. As such, we propose a ML pipeline for effective arousal detection. We trained our model with three publicly available data sets where we particularly focused on EEG and HRV data. Considering the scenarios, our proposed automated ML pipeline will overcome the model selection problem for arousal detection. Our trained ML model can be used for further development in VRET to overcome psychological distress in anxiety and fear-related disorders. As the first phase of work, we have implemented a biofeedback framework for VRET where we successfully provided feedback as a form of heart rate and brain laterality index from our acquired multimodal data for psychological intervention to overcome anxiety. Further useful applications of the model can be seen in meltdown moment detection in autism spectrum disorder (ASD) and other scenarios where stress and arousal play a significant role and early intervention will be helpful for physiological amelioration. For example, early identification and signalling of a meltdown moment can facilitate the initiation of targeted interventions preventing meltdowns, which will help parents, carers and supporting staff deal with such occurrences and reduce distress and harm in individuals with ASD. Finally, arousal and increasing stress have become buzzwords of recent times, adversely affecting a vast range of populations across the globe regardless of age group, ethnicity, gender, or work profile. Due to the long ongoing COVID-19 pandemic, changing scenarios, work patterns and lifestyles, increasing pressures, and technological advancements are a few possible reasons for this trend [56, 61, 81, 84]. Thus, accurate detection of distress-related arousal levels across the general population (e.g., in educational settings or the workplace) may help to avoid associated adverse impacts through effective interventions, prevent long-term mental health issues and improve overall well-being.

## Footnotes

1 https://physionet.org/content/eegmat/1.0.0/.
